# Personal

**Published:** 1914-12

**Authors:** 


### PERSONAL.

Announcement of removal, travel, and other matters of interest are requested. Please report errors in the listing of any physician in the State and other directories, that we may co-operate with the State Society in securing a correct list.

Dr. Isaac Sernoffsky announces his return from Vienna and location at 37 Allen Street, Buffalo. Practice confined to the eye.

Our Associate Editor, Dr. J. George Adami, of Montreal, has enlisted as a private in a battalion organized at McGill University. The true test of patriotism or devotion to any cause, is the willingness to be a private. A good share of the trouble in this world, from our early childhood up, is due to those who wont play unless they can be captain.

Dr. Raymond Sanderson of Canandaigua, formerly bacteriologist of Ontario Co., has accepted the same position for Ulster Co. with headquarters at Kingston. He is succeeded by Dr. Wm. A. Bing of Albany.

Mrs. Webber, Asst. Supt. of the Rochester Homoeopathic Hospital for seven years, has resigned to engage in foreign Red Cross Work.

Dr. George Leslie Miller of Niagara Falls has been appointed surgeon to Co. E., 3d Regt., with rank of second lieutenant.

Dr. E. C. Koenig of Buffalo has removed to 55 Park Street.

Dr. Lester I. Levyn announces his return from Europe and location at 203 E. Eagle Street, Buffalo. Practice limited to X-ray diagnosis and therapy.

Dr. Regina Flood Keyes of Buffalo sailed October 31, to serve in the American Ambulance Hospital of Paris.

---

The following Buffalo physicians have been appointed to collect funds for the relief of the Belgians: Dr. Irving M. Snow, chairman; Dr. E. S. Tobie, Dr. Park Lewis, Dr. George Critchlow, Dr. James Putnam, Dr. Arthur W. Hurd, Dr. Lee Masten Francis, Dr. John Pryor, Dr. C. R. Borzilleri, Dr. P. W. Van Peyma, Dr. Stephen Howell.

---

Dr. W. S. Grimes of Detroit has removed to the Market Building, Highland Park, Mich.

---

Dr. Robert Hebenstreit of Buffalo has moved to 1141 Genesee Street.

---

The automobile of Dr. Charles R. Cullinane of Buffalo was stolen a few days ago, but was found abandoned the next morning.

---

Dr. John D. Fowler announces the opening of his office at 101 Saratoga Avenue, Rochester.

---

Dr. Homer W. Smith announces the opening of his office at 247 Webster Avenue, Rochester.

---

Dr. James A. Gardner of Buffalo has been appointed to the consulting staff of the Bremerman Sanatorium, which will be erected at Potash Sulphur Springs, Lawrence, Ark. This will be the first institution in America for the exclusive sanatorial treatment of urologic cases.

